# Gene Sharing Yields an Enzyme with Two Binding Sites in One Subunit

**DOI:** 10.1371/journal.pbio.1000064

**Published:** 2009-03-31

**Authors:** Caitlin Sedwick

Complexity abounds in the natural world, within us and around us. Consider the human body. We exist thanks to the efforts of an estimated 50 trillion cells—nearly 100 times the number of stars in the Milky Way. Each cell performs a specialized function, conferred by the differential expression of the more than 25,000 protein-encoding genes in the human genome.

Proteins are complicated, too; they can act as enzymes (that accelerate specific chemical reactions), adhesins (that bind to other proteins and other large molecules such as complex carbohydrates and fats), structural elements (such as the cell cytoskeleton), and signal transducers. Multifunctional proteins perform some combination of these roles. But given the stringent physical and chemical demands associated with performing a given function, multifunctional proteins are typically composed of spatially distinct subunits (referred to as domains), each of which serves a separate biochemical function. For example, proteins with one subunit that has enzymatic function commonly link that enzyme subunit to one or more subunits that function as adhesins. It's highly uncommon, however, to find a single subunit of a protein that serves more than one purpose, for example, a subunit that acts as both an enzyme and as an adhesin. Yet such a dual function subunit is just what Cedric Montainer, Harry Gilbert, and colleagues describe in this issue of *PLoS Biology*.

To explore how single-subunit proteins might perform multiple functions, the authors focused on CE2 enzymes, a family of enzymes that help degrade plant cell walls. CE2 enzymes, esterases that split ester bonds in hemicelluloses, are part of the apparatus that some Clostridium bacteria use to chew through plant cell walls. CE2 enzymes target the acetyl groups attached to hemicellulose molecules that make a network with cellulose, the most abundant organic molecule on the planet. Cellulose is composed of long chains of glucose rings that, by interacting with each other through intramolecular and intermolecular hydrogen bonds, result in the formation of cellulose fibers. The abundant hydrogen bonding makes cellulose very difficult to attack enzymatically and gives plants their rigid structure. By contrast, hemicelluloses are less sturdy and are thus vulnerable to degradation—a job facilitated by CE2 enzymes.

Most CE2 family enzymes are single-subunit proteins, but one CE2 family member expressed by C. thermocellum, called *Ct* Cel5-CE2, is a multi-subunit protein. *Ct* Cel5-CE2 has a CE2 subunit (called the *Ct* CE2 domain) that shares a similar three-dimensional structure and amino acid sequence with other CE2 family proteins. However, when the *Ct* CE2 domain was first characterized, it was found to be a noncatalytic cellulose-binding subunit, rather than an esterase for hemicelluloses. This cellulose-binding feature of *Ct* CE2 was especially curious, because none of the other CE2 family members was known to bind cellulose, inspiring the authors to investigate whether the *Ct* CE2 domain also functioned as an esterase—which they have now discovered is indeed the case. The authors also confirmed that it could bind to cellulose, whereas other CE2 family members could not. Surprisingly, however, if they added both cellulose and hemicellulose together, the *Ct* CE2 domain could not act as an esterase on hemicellulose, because cellulose was bound to the *Ct* CE2 domain, therefore inhibiting its ability to act on hemicellulose. This observation spurred the authors to compare more carefully the structure of the *Ct* CE2 domain with that of other CE2 family members.

**Figure:**
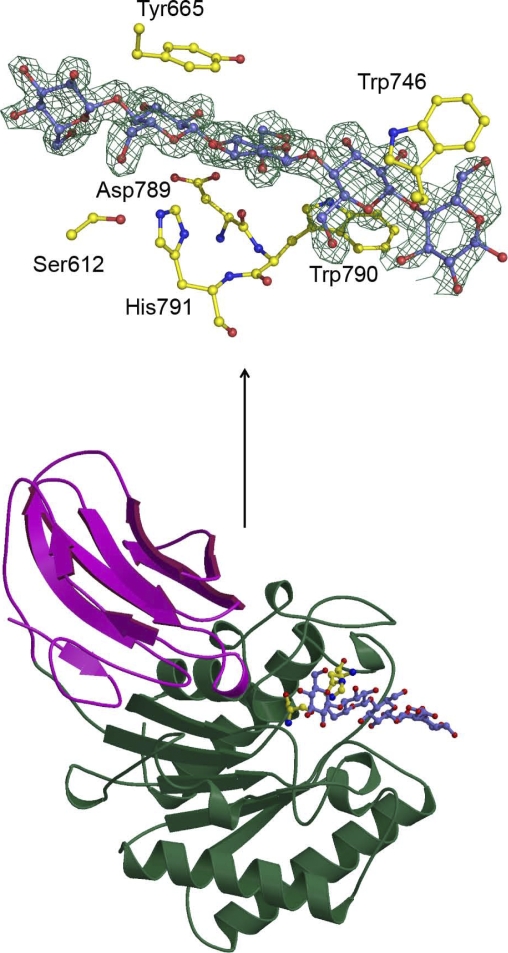
Shown here is *Ct* CE2 in complex with cellohexaose. The catalytic apparatus contributes to ligand recognition.

The structural studies showed something quite unusual: in the *Ct* CE2 domain, the binding site for cellulose exactly overlapped the enzymatic cleft. This suggests that a subunit that had first evolved as an esterase for hemicelluloses had acquired just the right geometric and sequence arrangement of amino acids to also allow binding to cellulose. None of the other CE2 family members shares quite the same sequence of amino acids with *Ct* CE2, which explains why they function solely as a hemicellulose esterase. The authors also show that one amino acid, Trp746, which is present in only one other CE2 family member (although in a slightly different geometric arrangement), is particularly important for *Ct* CE2 to bind cellulose.

The *Ct* Cel5-CE2 CE2 domain is unusual not just within its family but also among all other proteins. Cellulose binding by the CE2 domain appears to help augment the activity of a cellulase subunit found on another part of the protein, making the whole protein more effective at breaking down plant cell walls. This is a useful adaptation for C. thermocellum, as it helps the bacterium use the sugars in the cell wall as an important source of nutrients. And, understanding how this protein works may also be useful for humans looking to find better ways to crack plant cell walls to harvest biofuels.

The evolution of the *Ct* CE2 domain's cellulose-binding activity on top of its original hemicellulose esterase activity provides a striking example of dual functionality in a single domain. It's likely that there are other examples of proteins with dual functions executed by the same region. If so, proteins may be even more complicated than previously believed.

